# Vision in the margins: the association between census tract neighbourhood disadvantage and visual difficulty and blindness in the United States

**DOI:** 10.1038/s41433-026-04515-z

**Published:** 2026-05-15

**Authors:** Jenna Hart, Minnie Bluhm, Tiffani R. Spaulding, Amanda Nwokeji, Angela R. Elam, Dean VanNasdale, Erica Shelton, Maria A. Woodward, Paula Anne Newman-Casey, Patrice M. Hicks

**Affiliations:** 1 Wayne State University School of Medicine, Detroit, MI USA; 2 Department of Health Sciences, Eastern Michigan University, Ypsilanti, MI USA; 3 Department of Ophthalmology and Visual Sciences, University of Chicago, Chicago, IL USA; 4 University of Michigan Medical School, Ann Arbor, MI USA; 5 Department of Ophthalmology and Visual Sciences, University of Michigan, Ann Arbor, MI USA; 6 Institute for Healthcare Policy and Innovation, University of Michigan, Ann Arbor, MI USA; 7 School of Optometry, The Ohio State University, Columbus, OH USA; 8 Department of Health Behavior and Health Equity, School of Public Health, University of Michigan, Ann Arbor, MI USA

**Keywords:** Risk factors, Epidemiology

## Abstract

**Background/Objectives:**

To investigate the utility of using a census tract (CT) measure to assess neighbourhood disadvantage and to explore its effects on CT measures of visual difficulty and blindness (VDB).

**Subjects/Methods:**

This cross-sectional study used census tract data from the 2018 to 2022 American Community Survey and the National Neighbourhood Data Archive Neighbourhood Socioeconomic Status and Demographic Characteristics. CT measures of neighbourhood disadvantage, VDB, and demographics were summarized with descriptive statistics (mean, standard deviation (SD)). The main outcome was the number of CTs residents reporting VDB and the association with neighbourhood disadvantage (an aggregate measure ranging from 0 to 1 with higher scores indicating more disadvantage) assessed using logistic regression.

**Results:**

In total, 83,388 CTs were included, with a mean of 2.53% of the population reporting VDB (SD = 2.62) and a mean neighbourhood disadvantage of 0.18 (SD = 0.12). Neighbourhood disadvantage was associated with 2.9% increased odds of VDB (Odds Ratio (OR): 1.029; 95% Confidence Interval (CI): 1.029,1.029; *p* < 0.001), after adjusting for neighbourhood demographics including median age, percentage of the CT that were female sex, percentage of the CT that identified as a person of colour, population size of the CT, and the state in which the CT was located.

**Conclusion:**

This study identified that neighbourhood disadvantage was associated with a greater number of residents reporting VDB. Clinicians could use this measure to identify neighbourhoods with both higher levels of visual impairment and greater neighbourhood deprivation, allowing for targeted interventions that provide eye disease screening and eye care to those most at risk for poor vision outcomes.

## Introduction

Visual impairment, defined as living with visual acuity loss or blindness, impacts the health of our communities impacting quality of life by interfering with one’s ability to work, read, drive, navigate routine tasks, and maintain independence [1–3]. Vision loss is associated with negative health outcomes such as depression, diabetes, falls, stroke, cognitive decline, and even premature death. People fear the serious consequences of vision loss. In a survey of over 2000 adults, nearly half of the respondents rated losing vision as the worst possible health outcome—even worse than hearing loss or losing a limb [1, 4]. A recent meta-analysis suggests more than 7 million people in the United States are visually impaired, while other estimates approach 21 million [1, 5, 6]. Visual impairment poses major economic burdens. In the United States, direct and indirect costs, which include costs due to loss of productivity, have been estimated at more than $134 billion [7].

Modifiable health factors are associated with visual impairment [8]. Americans living in areas with fewer economic and social resources have had higher rates of visual impairment and less eye care utilization when compared with those living in areas with more resources [9–11]. Since visual impairment can be mitigated with early detection and treatment of ocular disease, it is important that those at risk have access to care; however, poorly resourced groups often lack facilitators to eye care access—such as money, transportation, private insurance, childcare, eldercare, or local clinics—and are therefore less likely to receive timely ophthalmic care, resulting in worse visual outcomes [12].

Ocular diseases that lead to visual impairment are not evenly distributed across geographic regions and may be seen at a higher prevalence in areas with more deprivation (worse education, lower income, poorer housing quality, or higher unemployment) [13, 14]. Disease severity for people with diabetic retinopathy is related to residing in an area with greater neighbourhood deprivation [15]. Patients living in areas with greater deprivation were both farther from eye clinics and more likely to develop proliferative retinopathy [16]. Among patients with microbial keratitis, the odds of presenting with best corrected visual acuity worse than 20/40 is higher among those living in areas with greater deprivation compared to patients in areas of less deprivation [17].

The Neighbourhood Atlas’s Area Deprivation Index (ADI), a widely used metric in eye and vision research, is specifically designed for use with census block group data and is not validated for census tracts or ZIP codes [16–25]. Block groups and tracts are geographic units defined by the U.S. Census Bureau for analysis. A census block group consists of several contiguous blocks and typically contains between 600 and 3000 people. In contrast, a census tract is a larger unit, usually encompassing 1200 to 8000 people, with an optimal population of about 4000. At the smallest scale, a census block is a single unit within a block group (e.g. a city block) [26]. Unlike these Census-defined areas, ZIP codes are created by the U.S. Postal Service for mail delivery and do not represent population-based boundaries [27, 28]. In this study, we evaluated the utility of a National Neighbourhood Data Archive’s measure called ‘Neighbourhood Disadvantage’ reported at the census tract and its relationship to visual difficulty and blindness (VDB)—a measure collected by the American Community Survey and also reported at the census tract [28].

## Methods

### Ethical review

The University of Michigan Medical School classified this cross-sectional study as exempt since it involves secondary analysis of publicly available data. The study complied with the Declaration of Helsinki.

### Data

This study used two data sources: the National Neighbourhood Data Archive (NaNDA) and the American Community Service Survey (ACS). The exposure variable was ‘neighbourhood socioeconomic disadvantage’, which was sourced from the NaNDA, with 5-year data from 2018 to 2022 at the census tract level. NaNDA is an open data repository, created by researchers in the Social Environment and Health Programme at the University of Michigan Institute for Social Research, provides publicly available datasets to advance research on the relationship between neighbourhoods and health. The NaNDA ‘neighbourhood socioeconomic disadvantage’ is a mean of three ACS 2018–2022 key economic indicators including proportion of families with incomes less than $40,000 United States Dollars (USD), proportion of people with annual income of below the poverty level, and proportion of households receiving public assistance income or food stamps [29–31]. The measure is available for ACS 2008–2012, 2013–2017, and 2016–2020, and 2018–2022. The 2016–2020 utilizes the same variables as the 2018–2022 measurement utilized for this analysis. The ACS 2008–2012 and 2013–2017 variable is a mean of five ACS measures including families below the poverty line, families receiving public assistance income, adults unemployed, female headed families, and non-Hispanic Black residents [32]. The update was made starting with the 2016–2020 data because the previous ACS measures have become less strongly linked to neighbourhood disadvantage over time, indicating that economic factors now primarily drive disadvantage. This measure is also available at the zip code level use when eye and vision outcomes may not be available at the more granular census tract level. The measure is an aggregate measure and ranges from 0 to 1, where increasing values mean more disadvantage [33].

The outcome variable was derived from the Visual Difficulty and Blindness (VDB) data in the 2018–2022 American Community Survey (ACS). The ACS accommodates individuals with vision impairment participating in ACS through either telephone or face-to-face interviews conducted by field staff. The ACS surveyed the civilian, noninstitutionalized population who self-reported VDB by responding to the query, “Are you blind, or do you have serious difficulty seeing even when wearing glasses?” [30]. ACS then uses responses to within a census tract to create a census tract-level prevalence of VDB.

**Fig. 1:**
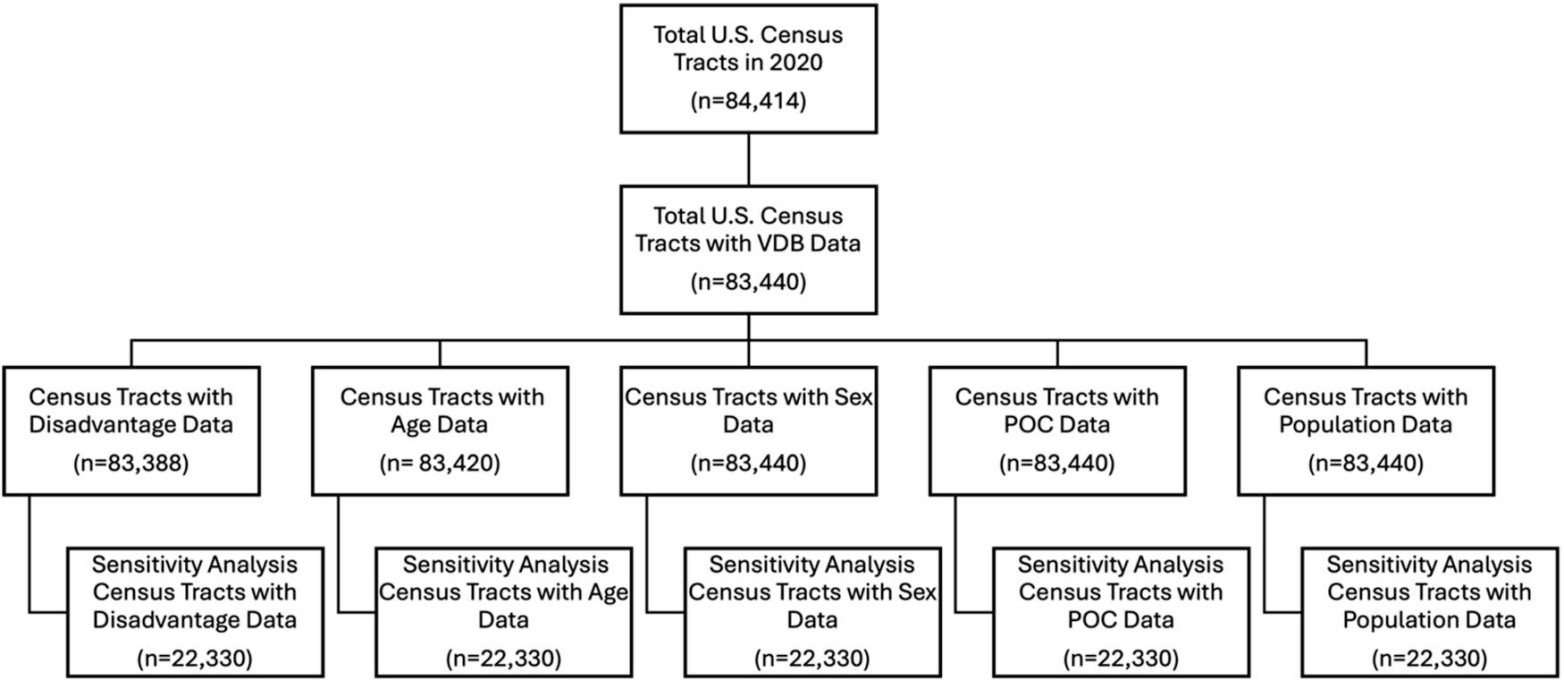
STROBE figure for disadvantage.

The demographics from the 2018 to 2022 ACS census tract data was comprised of the median age, the percentage of residents who identified as female, the percentage of residents identifying as belonging to a racial or ethnic minority group, the population size of the census tract, and the state in which the census tract is located [31–34]. Data on visual difficulty/ blindness, and census-tract demographics were sourced from PolicyMap [35]. Margins of error for the variable VDB were obtained from the Census website [36–40]. The study followed the Strengthening the Reporting of Observational Studies in Epidemiology (STROBE) guidelines (Fig. 1).

### Statistical analysis

The primary analysis examined all census tracts that had a VDB measure. The sensitivity analysis excluded census tracts considered unreliable, specifically those with a coefficient of variance over 40% for the VDB measure, calculated as (standard error (SE)/estimate) × 100. Census tract measures of neighbourhood disadvantage, VDB, and demographics (age, sex, race/ethnicity, and population size) were summarized with descriptive statistics (mean, standard deviation (SD), median, and interquartile range (IQR)). Univariate associations with a continuous measure of VDB were evaluated using Pearson’s chi-squared test (r). Logistic regression models were used to evaluate the relationship between measures of disadvantage in census tracts and the likelihood of VDB events. The methodology of transforming the prevalence of VDB at the Census Tract-level reported by ACS to the probability of VDB has been previously described. We utilized this approach to estimate the probability of VDB in each census tract by calculating the frequency of VDB events relative to the population size (events/trials) [41–43]. The model results for census tract disadvantage are reported with odds ratios (ORs), 95% confidence intervals (CIs), and two-sided P-values, with significance set at *P* < 0.05. The multivariable logistic regression models were adjusted for demographic factors within the census tracts, including median age, percentage of the population identifying as female sex, the logarithm of the percentage of the population identifying as a person of colour (to account for skewness), the logarithm of population size (to account for skewness), and state. These variables were selected for model adjustment based on previously published literature on neighbourhood inequities and health (Supplementary Fig. 1) [42–44]. Statistical analyses were performed using R, version 4.4.2 (R Foundation for Statistical Computing).

## Results

Data analysis was completed in February 2025. According to the census, the 2020 tract designations include 84,414 census tracts, of which 83,440 (98.85%) had VDB data available (Fig. 1). Of those with measures of VDB, 83,388 (99.94%) of census tracts had measures of neighbourhood disadvantage (Table 1) which were included in the overall analysis. Mean census tract disadvantage was 0.18 (SD = 0.12) ranging from 0 to 1 (Interquartile Range: 0.10–0.24). In the census tracts, the mean population size was 3965 people (SD = 1706 people), the mean age was 40.12 (SD = 8.25) years, the mean percentage of females was 50.43% (SD = 4.97%), and the mean percentage of racial and ethnic minority individuals was 40.35% (SD = 29.50%) (Table 1). Increased prevalence of VDB was moderately associated with disadvantage by census tract (r = 0.39; 95% CI: 0.38–0.39; *p* < 0.001). VDB was only minimally correlated with demographic factors by census tract percentage of the population that identified as members of a racial or ethnic minority group (r = 0.10; 95% CI: 0.09, 0.11; *p* < 0.001), age (r = 0.08; 95% CI: 0.08, 0.09; *p* < 0.001), and the percentage of the population that was female (r = 0.03; 95% CI: 0.02, 0.03; *p* < 0.001), and decreased population size (r = –0.15; 95% CI: 0.882, 0.885; *p* < 0.001). (Table 1). In total, 22,330 (26.5%) census tracts were included in the sensitivity analysis (Fig. 1). Similar associations were observed in the sensitivity analysis (Table 1).

**Table 1:** Descriptive statistics of census tract–level variables and sensitivity analysis.

Variable	Census Tracts, No.	Mean (SD)	Median (IQR)	Pearson correlation coefficient (95% CI)	*P* value
VDB, %	83,440	2.53 (2.62)	2.01 (1.08–3.38)	—	—
Age, y	83,420	40.12 (8.25)	40.00 (35.00–45.00)	0.08 (0.08–0.09)	<0.001
Population, No.	83,440	3965 (1706)	3776 (2741–4957)	−0.15 (−0.16–0.15)	<0.001
Racial and ethnic minority individuals, %	83,440	40.35 (29.50)	32.74 (14.86–62.99)	0.10 (0.09–0.11)	<0.001
Females, %	83,440	50.43 (4.97)	50.44 (47.89–53.10)	0.03 (0.02–0.04)	<0.001
Neighbourhood Disadvantage	83,388	0.18 (0.12)	0.16 (0.10–0.24)	0.39 (0.38–0.39)	<0.001
**Sensitivity Analysis—Coefficient of Variation Analysis (40%)**					
VDB, %	22,330	3.93 (2.44)	3.38 (2.32,4.83)	—	—
Census Tract Disadvantage	22,330	0.22 (0.12)	0.19 (0.13–0.28)	0.44 (0.43, 0.46)	<0.001
Age, y	22,330	41.04 (8.06)	41.00 (36.00,46.00)	0.02 (0.01, 0.04)	<0.001
Population, No.	22,330	4034 (1626)	3890 (2868,4953)	–0.22 (–0.23, 0.21)	<0.001
Racial and ethnic minority individuals, %	22,330	37.59 (30.58)	27.86 (10.58,60.80)	0.19 (0.17, 0.20)	<0.001
Females, %	22,330	50.17 (5.08)	50.33 (47.93,52.85)	0.04 (0.03, 0.05)	<0.001

*y* years, *No.* number.

Logistic regression models assessing the association between census tract measures of disadvantage and VDB are presented in Table 2. In the univariate model, a 0.01-unit increase in the neighbourhood disadvantage (more disadvantage) was associated with 2.6% increased odds of VDB (OR:1.026; 95% CI: 1.026, 1.026; *p* < 0.001). After adjusting for census tract demographics, a 0.01-unit increase in the neighbourhood disadvantage was associated with 2.9% increased odds of VDB (OR:1.029; 95% CI: 1.029,1.029; *p* < 0.001). The sensitivity analysis (Table 2) showed an 0.01-unit increase in the neighbourhood disadvantage was associated with an 2.2% increased odds of VDB (OR:1.022; 95% CI:1.022, 1.022; *p* < 0.001) and remained statistically significant after adjusting for census tract level demographics (OR: 1.021; 95% CI: 1.020, 1.021; *p* < 0.001). Both the unadjusted and adjusted neighbourhood deprivation OR remained statistically significant, but it declined in magnitude.

**Table 2:** Association between neighbourhood disadvantage with the probability of vision difficulty and blindness and sensitivity analysis.

Variable	Unadjusted Univariate Model		Adjusted Multivariable Model*	
	Odds ratio (95% CI)	*p* value	Odds ratio (95% CI)	*p* value
Neighbourhood Disadvantage (per 0.01-unit)	1.026 (1.026,1.026)	<0.001	1.029 (1.029,1.029)	<0.001
Age, y	1.008 (1.008,1.008)	<0.001	1.023 (1.023,1.023)	<0.001
Population, No.*^	0.720 (0.718,0.721)	<0.001	0.972 (0.971,0.974)	<0.001
Racial and ethnic minority individuals, %^	1.108 (1.106,1.109)	<0.001	1.018 (1.015,1.020)	<0.001
Females, %	1.006 (1.006,1.006)	<0.001	0.998 (0.998,0.998)	<0.001
**Sensitivity Analysis–Coefficient of Variation Analysis (40%) Logistic Regression Modelling**				
Census Tract Disadvantage (per 0.01-unit)	1.022 (1.022, 1.022)	<0.001	1.021 (1.020, 1.021)	<0.001
Median Age, y	1.002 (1.002, 1.002)	<0.001	1.014 (1.013, 1.014)	<0.001
Population, No.^	0.701 (0.699, 0.703)	<0.001	0.846 (0.844, 0.849)	<0.001
Racial and ethnic minority individuals, %^	1.214 (1.211, 1.217)	<0.001	1.104 (1.100, 1.108)	<0.001
Females, %	1.005 (1.005, 1.006)	<0.001	0.999 (0.999- 0.999)	<0.001

*Adjusted for median age, logarithm of the population size, percentage of the population that were female, percentage of the population that identified as a racial or ethnic minority group, and state. ^Logarithm of variable.

## Discussion

In this cross-sectional study, we used a census tract measure of neighbourhood disadvantage and found that at the census tract level, which is a larger geographical area than the census block level, neighbourhood disadvantage is associated with VDB. This study found that a 0.01 unit increase in neighbourhood disadvantage was associated with 2.9% increased odds of VDB, even accounting for demographic factors including median age, percentage that were female sex, percentage that identified as a person of colour, population size of the census tract, and the state in which the census tract was located. (Table 2). These findings persisted within the sensitivity analysis that adjusted for the same neighbourhood demographics, though the magnitude declined (Table 2). The findings from this study are consistent with previous work that indicates that neighbourhood disadvantage measured at the census block level is associated with poor eye and vision health outcomes.

Neighbourhood deprivation, as measured by the Area Deprivation Index (ADI), has been linked to various eye diseases and vision and eye outcomes [16, 19, 45]. Awidi et al. found that patients who presented for cataract surgery had worse presenting visual acuity among those living in higher national ADI quartiles (more deprived areas) compared to those in more affluent communities (national ADI quartile 4 (highest level of deprivation) with patients testing one line worse on average; β = 0.95, *p* < 0.001) [21]. Similarly, patients presenting with microbial keratitis had 30% increased odds of worse visual acuity (< Snellen 20/40) for every 10-unit higher national ADI (OR:1.30 per 10-unit increase; 95% CI:1.25–1.35; *p* < 0.001) [17]. In addition, a 1-unit increase in state ADI was associated with worse presenting glaucoma severity, as measured by mean deviation (β = −0.31, 95% CI = −0.41 to −0.22, *P* < 0.001) [46]. Among patients with rhegmatogenous retinal detachments, for every 10-point increase in mean neighbourhood ADI, patients had 14% increased odds of presenting vision worse than 20/40 (OR:1.14; 95% CI:1.04–1.24; *p* = 0.004) and a 13% increased odds of having detachments involving the fovea (OR: 1.13; 95% CI: 1.04–1.22; *p* = 0.005) [45]. Deprivation is also associated with delays in care. There was a 68% increase in time to glaucoma surgery among those living in areas with the highest national deprivation quartile compared to the least (national ADI quartile 4; Time Ratio: 1.68; 95% CI: 1.20–2.36; *p* = 0.002) [47].

While ADI can be an effective tool to understand neighbourhood deprivation, it may not be applicable in all clinical and research settings. ADI approximates to very small geographic areas i.e. the census block group (never crossing geographic boundaries of state, county or tract), for which it is considered valid by the Neighbourhood Atlas. But linking patient or resident information at this geographic level may not be feasible. Patient or resident data might not be available or collected at the census block group level. Electronic health records or residential addresses are typically available at larger scales, such as census tracts. In addition, granular geographic information can raise privacy concerns, especially when dealing with rare eye conditions. When block group data is unavailable, using data at the census tract level—such as NaNDA neighbourhood disadvantage scores—provides a way to localize patient geographic information.

Socioeconomic components of neighbourhood disadvantage impact visual impairment [48]. Patients with lower incomes, as defined by poverty income ratio, are less likely to visit the eye clinician (*p* < 0.05) and more likely to experience difficulties paying for eyeglasses (*p* < 0.001) when compared with those who have higher incomes in an investigation of Centres for Disease Control and Prevention’s National Health Interview Survey data [49]. People making less than $35,000 USD in annual income have greater odds of severe visual impairment (OR: 1.22; 95% CI:1.10–1.34; *p* < 0.001) [50]. Su et al. demonstrated visual difficulty was associated with having health care coverage through Medicaid, a public assistance programme, (OR:1.44; 95% CI:1.23–1.67; *p* < 0.05), and financial difficulty in obtaining medication (OR:1.72; 95% CI:1.54–1.93; *p* < 0.05) [51].

Future research could explore if only certain components of the NaNDA measure of neighbourhood disadvantage—family annual family income <$40,000 USD, family receiving public assistance income, and family living below the poverty line, affect VDB in an individual. There may be a more nuanced element of neighbourhood disadvantage and VDB that would be amenable to targeted interventions or updated policies. Similarly, qualitative studies with patients with eye diseases could provide insights into the relationship between experiences, perceptions, neighbourhood factors, and eye care and vision health outcomes. Furthermore, the components of the NaNDA measure have shifted over time, with economic factors now playing a greater role than earlier social indicators—such as female-headed households, non-Hispanic Black race, unemployment, and poverty—following a re-evaluation of the NaNDA socioeconomic measures [32]. It will be important to monitor the evolution of the underlying components of neighbourhood disadvantage so that screening approaches and interventions can be adapted as needed.

This study has limitations. First, the study data is from 2018-2022 at the census tract, as the data was obtained from 5-year ACS and the NaNDA measure of neighbourhood disadvantage, which was the most recent years available for the NaNDA measure [52]. Thus, the findings of this study are only generalizable for this timeframe. Future research should evaluate if these associations remain true in subsequent data updates. Second, the vision data from the ACS is by patient self-report, not clinical visual acuity testing, so there may be social-desirability bias. Third, census tracts with missing VDB measures were excluded, thus the study findings may not generalizable to those census tracts.

This study found an association between VDB and neighbourhood disadvantage, at the census tract, consistent with studies using census block (i.e., area deprivation index). The NaNDA Neighbourhood Disadvantage measure can inform clinicians about the neighbourhoods of the populations they serve in the clinic, helping identify areas with a higher disadvantage score and likelihood of VDB. It can also guide epidemiologists and policymakers in targeting areas for vision-related interventions. Researchers can use this measure of census-track or zip code neighbourhood disadvantage in lieu of census block geographic levels to match neighbourhood-level data from different sources. The impacts of ocular disease can often be mitigated with early detection and treatment. Findings from this study suggest neighbourhood disadvantage measures at the census tract level can inform a targeted approach to screen those most at risk for VDB and identify those who can benefit from early detection and treatment, leading to improved eye health and vision outcomes.

Supplemental material is available at Eye’s website.

## Summary

### What was known before

- The Area Deprivation Index (ADI) is associated with eye disease. ADI is associated with microbial keratitis. ADI is also associated with screening positive for glaucoma.
- ADI is only verified for use at the census block group. ADI cannot be used for data where patient address can only be geocoded to the zip code. Similarly, it cannot be used for data where patient address can only be geocoded to the census tract.

### What this study adds

- The National Neighbourhood Data Archive Neighbourhood Disadvantage measure is useful for eye and vision studies. This measure is available for both the census tract and zip code, which is useful for eye care and vision health data only available at these geographic levels as ADI is not validated at these geographic levels.
- Neighbourhood disadvantage is associated with vision difficulty and blindness. Neighbourhood disadvantage was associated with 2.9% increased odds of VDB (Odds Ratio (OR): 1.029; 95% Confidence Interval (CI): 1.029,1.029; *p* < 0.001). This positive association was observed even after accounting for neighbourhood demographic measures.

## Supplementary information

Supplemental Figure 1. Conceptual Model of the association between Census Track Area Deprivation Index and Visual Difficulty and Blindness

## Data Availability

All data comes from publicly available sources. The datasets generated during and/or analysed during the current study are available from the corresponding author on reasonable request.
