# Supplementary material for: Vision in the margins: the association between census tract neighbourhood disadvantage and visual difficulty and blindness in the United States
Source: Eye (Lond). 2026 May 15;40(11):1734–9. doi: 10.1038/s41433-026-04515-z (PMC13416027; doi:10.1038/s41433-026-04515-z)
Supplement: Supplementary file 1 — Supplemental Figure 1. Conceptual Model of the association between Census Track Area Deprivation Index and Visual Difficulty and Blindness [file 41433_2026_4515_MOESM1_ESM.docx]

Supplemental Figure 1. Conceptual Model of the association between Census Track Area Deprivation Index and Visual Difficulty and Blindness
